# Physiological Observations on the Transformations of the Organs of the Human Body

**Published:** 1806-11

**Authors:** C. L. Dumas

**Affiliations:** the Imperial Institute, Professor in the School of Medicine at Montpellier, &c. &c.


*Physiological Observations on the Transformations of the Organs of the Human Body. By* C. L. DUMAS, *of the Imperial Institute, Professor in the School of Medicine at Montpellier, &c. &c.*

( Continued from our last, pp. 301—304. )

In neither case, however, is the organ so changed, as to acquire a texture similar to that of another organ. In the first case, the transition to a gelatinous state, ought rather to be considered as a degeneracy than a transformation of the organ; in the second, gelatino-mucous bodies are spontaneously produced, without the texture of any organ having suffered the least change. We may notwithstanding mention as a proof of the transformation of certain organs, by the predominance of gelatinous matter, that change by which the bones, and particularly the flat bones, are converted into a soft, flexible substance, similar to that of the mucous membranes. Fernelius, Hildanus, and Thomas Bartholin, &c. mention instances of this kind, in which, according to the expression of Morgagni, the bones were changed into a red coloured flesh, wholly destitute of fibres. *In carnem non fibrosam quidem sed rubicundam conversa.* There is recorded in the History of the Royal Academy of Sciences, for 1700, an extraordinary case of a woman, in whom all the bones, the teeth excepted, became so gelatinous as to appear only a soft, fleshy, and fungous mass. I have seen, in the possession of a celebrated Surgeon, the patella attenuated, and reduced to the nature of the tendons, so as not to be distinguishable from the tendinous extremities of the muscles attached to it. The conversion of bones into a cartilaginous state is too well known and established, to render farther examples of the fact necessary.

The conversion of the bones into membranes, flesh, and cartilage may depend on two causes.

1. On a superabundance of gelatine supplied by the blood.

2. On the loss of a portion of the calcareous earth, or the earthy saline matter, on which their solidity depends.

Each of these causes may act separately, or in conjunction, in producing this effect. It is very difficult to determine, when the conversion in question proceeds from a deficiency of calcareous earth, or from an excess of gelatinous matter.

Earthy calcareous salts, when existing in too great a quantity in the animal body, may be carried to different organs, and render them equally hard, as those parts naturally possessing the greatest degree of solidity. There is no part, or organ, which is not liable to suffer this change. Even the brain itself, under certain circumstances, becomes ossified; but nothing is more common than the transition of the cartilages, the ligaments, the membranes, and even of the large vessels, into an osseous state. It is not unusual for the articular cartilages to acquire the hardness of bone at a very advanced period of life. The bones themselves not unfrequently unite, and become anchylosed at their articulations, by the induration and ossification of the cartilages in some old men, who had long laboured under diseases of the joints, which forced them to remain in a state of immobility, or absolute rest. The dura mater is also frequently found ossified throughout a considerable portion of its extent. The arteries, which proceed to the brain, sometimes display a singular aptitude to ossify. The aorta, the carotids, the hepatic, splenic, and other arteries, are often found in a state of complete ossification. The heart likewise displays indurated and ossified points, not only on its exterior but interior surface. The pylorus, or inferior orifice of the stomach frequently contracts a degree of solidity approaching to that of the bones. The external membranes of the spleen and liver, also occasionally acquire, in consequence of disease, the hardness of bones. The tendinous extremities of the muscles likewise assume an ossific character, in consequence of which small bones are formed that occupy the interval between the articular surfaces. The conversion of certain parts into a bony state is not the only effect produced by the predominance, or undue distribution of the saline earthy matter. The membranes, arterial vessels, muscles, and the different viscera, without being absolutely changed into bone, acquire a cartilaginous character from the deposition of too great a quantity of earthy salts. Such conversions, in consequence of an excess of calcareous earth, are greatly assisted by the waste or or destruction of the gelatine, albumen, and other substances, with which the earthy salts are always combined in the animal body; but it is always ultimately to a predominance of calcareous matter that we must attribute the conversion of an organ into a cartilaginous and osseous state.

In this way may be also explained the conversion of certain parts into a corneous substance, or into a matter similar to that of the nails. To the same cause must be attributed the formation of those scales and solid protruberances with which the external integuments of individuals are sometimes affected; this phenomenon, it is evident, must necessarily be referred to that class of transformations, which depend on an excess and undue distribution of calcareous earthy salts.

After having examined the various transformations of which the organs of the human body are susceptible in their physical constitution, and in their chemical composition, it next remains for us to enquire, how these organs sometimes experience such changes in their structure and functions, as to produce in them the organic arrangement, or the vital operations which are characteristic of other organs. In this inquiry, it is first necessary to ascertain what we ought to understand by the structure of an organ, and under what different points of view we should contemplate this structure. In the organization of the human body, and of its parts, two very distinct things must be considered. First, the external and evident conformation, which is founded on the assemblage, the relation, and the connection of all the parts subservient to the formation of the organs. Second, the internal order and disposition of the most obscure parts, which determine the nature and character of their organic texture. The alterations capable of transforming the structure of the organs are also of two kinds; viz. those which only change the order and distribution of the parts necessary to the conformation of any member, or organ; and those which essentially affect the arrangement and disposition of the parts, on which their peculiar organization depends.

The first species of alteration is so evident as not to require the same difficult and delicate researches as the second, which is of a much more minute and complicated nature. In this order must therefore be placed those changes which occur in the structure of organs, or members in consequence of some remarkable alteration in the number, position, form, relations, assemblage, and con-

 nection nection of the parts of which they are composed. Thus the addition or suppression of an osseous portion, the reciprocal change of different cavities and protuberances, the displacement of the nerves, muscles, and vessels by which their relative situation is deranged, are among the principal alterations which affect the external structure of the human frame, and which, in some instances, seem to produce a complete transformation of the organs. The external members, which serve as the instruments of locomotion, are especially subject to such changes, because they are composed of several distinct and detached portions; the position and relative situation of which may be singularly varied.

The viscera contained in the large cavities, are most liable to alterations in their inmost structure. Both the viscera, and the members, however, often suffer from these two species of transformation, examples of which are furnished by every part of the human body. The distribution of arteries, veins, and nerves, the situation of eminences, curvatures, orifices, and valves, the correspondence of projections, angles, and cavities, &c. are all subject to variation in the abdominal and thoracic viscera. In this way the conformation of a viscus, such as the stomach being changed, may become analogous to that of another wholly different.

In some subjects, the ductus choledochus communis, instead of entering the duodenum, opens into the stomach, while, in others, the valvula pylori is not in its natural situation, but attached to the superior extremity of the duodenum, so that this intestine and the stomach appear as if they had undergone a reciprocal transposition. The inferior portion of the colon, sometimes exhibits the folds and the valves so conspicuous in the jejunum.

An appendix similar to that of the colon is frequently met with in the duodenum, ilium, and rectum. Very singular changes likewise occur in the vessels and nerves of the organs contained in the same cavity. Thus the nerves, and the vessels of the stomach, liver, spleen, urinary bladder, kidneys, and mesentery, are frequently found intermingled, and occupying each others place. We once observed the ureters running in a contrary direction to that which they usually take, so that they appeared to originate from the bladder, and to terminate in the kidneys.

The works of anatomical writers abound with examples of similar degenerations in the different viscera. I have here only noticed such a number, as may suffice to establish lish the character of the class to which they belong. The following examples do not regard the structure of the viscera, but the conformation of the members.

In the Ephemerides Curiosorum Naturæ, mention is made of several articulations, in which the protuberances and corresponding cavities of the bones were found to have changed their respective places. It is well known, that, in some cases of fractures, the ends of the bones do not unite, but become smooth and round, and move readily upon each other, giving rise to what is commonly termed an artificial articulation. The action of the musculi rotatores of the thigh and arm, is sometimes so great, as to flatten the large tuberosities of the humerus and femur, so that in rickety subjects, the difference of these two bones, at their upper extremity, cannot be distinguished.

( To be Continued. )

